# International consensus-based ranking of definitions for poor response to primary total knee arthroplasty: a Delphi study

**DOI:** 10.1007/s00402-024-05515-y

**Published:** 2024-09-11

**Authors:** Malou E.M. te Molder, Stefaan van Onsem, José M.H. Smolders, Michelle M. Dowsey, Ola Rolfson, Jasvinder A. Singh, Marinus de Kleuver, Petra J.C. Heesterbeek, Cornelia H.M. van den Ende

**Affiliations:** 1 Department of Research, Sint Maartenskliniek, P.O. box 9011, Nijmegen, 6500 GM The Netherlands; 2 Department of Orthopedic Surgery, Radboud University Medical Center, Nijmegen, The Netherlands; 3 Department of Orthopaedics and Traumatology, AZ Alma, Eeklo, Belgium; 4 Department of Human Structure and Repair, Ghent University, Ghent, Belgium; 5 Department of Orthopaedic Surgery, Sint Maartenskliniek, Nijmegen, The Netherlands; 6 Department of Surgery, University of Melbourne, Fitzroy, Victoria Australia; 7 Department of Orthopaedic Surgery, St Vincent’s Hospital, Fitzroy, Victoria Australia; 8 Department of Orthopedics, Institute of Clinical Sciences, The Sahlgrenska Academy, University of Gothenburg, Gothenburg, Sweden; 9 Medicine Service, Birmingham Veterans Affairs Medical Center, Birmingham, Alabama USA; 10 Department of Medicine at the School of Medicine, University of Alabama at Birmingham, Birmingham, Alabama USA; 11 Department of Epidemiology, School of Public Health, University of Alabama at Birmingham, Birmingham, Alabama USA; 12 Department of Rheumatology, Radboud University Medical Center, Nijmegen, The Netherlands

**Keywords:** Total knee arthroplasty, Definitions, Poor response, Delphi study

## Abstract

**Introduction:**

This study aimed to rank definitions for measuring poor response one year after TKA, after assessing the face validity and feasibility of existing or newly proposed definitions.

**Materials and methods:**

An international, three-round, online modified Delphi study was conducted with sixty-nine panelists from twenty-three countries. Definitions were derived from a literature review or were newly proposed by an expert group. Panelists rated the face validity and feasibility of definitions, and could propose additional new definitions in round 1. Panelists reconsidered their rating of existing definitions, and rated newly suggested definitions (round 2). Definitions with a median score for face validity < 6.5 were removed from the list, and panelists distributed 100 points among the remaining definitions for ranking (round 3).

**Results:**

Fifty-one panelists completed all three rounds (response rate 74%), and the prioritized list of definitions in round 3 comprised seventeen definitions. The single-item definition of (dis)satisfaction with the outcome of TKA obtained the highest scores for face validity and feasibility (7.5, and 8.5 respectively), and the definition “No improvement in pain OR daily knee functioning compared to pre-operative status” was the highest prioritized. In general, definitions reflecting change from the perception of patients were higher ranked than definitions requiring both preoperative and postoperative assessment of validated questionnaires.

**Conclusions:**

This study identified seventeen potential definitions of poor response to TKA, offering valuable options for integration into quality assessment investigations. Remarkably, all identified definitions were patient-centered and none were clinician-centered. Single-item questions, capturing change from the patient’s viewpoint, appear to be the most practicable format to assess response.

**Supplementary Information:**

The online version contains supplementary material available at 10.1007/s00402-024-05515-y.

## Introduction

Approximately 10 to 20% of patients undergoing total knee arthroplasty (TKA) report unsatisfactory outcomes, characterized by persistent pain, inadequate enhancements in physical functioning, and/or unfulfilled expectations [1, 5, 8].

To effectively address and mitigate these dissatisfaction rates, a definition of poor response to TKA is needed. This definition could serve as a foundational element for initiating an actionable quality improvement cycle. A variety of dichotomous definitions of poor response to TKA comprising one or more different dimensions of outcome have been described in the literature [15], to quantify the proportion of patients with poor response to TKA. This large variety of definitions impedes the comparisons of poor response to TKA over time and across hospitals and countries. The need for a multidimensional combination of outcome domains (e.g. pain and function) has been recognized to describe failure (i.e. poor response) after TKA [12], but to date, an internationally accepted definition with good performance for measuring poor response to TKA is lacking [17].

A comprehensive definition of poor response to TKA after one year should encompass specific criteria that identify patients with an unfavorable course. This definition must outline the domain(s) or outcome measures indicative of poor response detailing criteria concerning both the extent and nature of change. This could involve relative or absolute changes compared to preoperative status or the establishment of a postoperative threshold beyond which patients are deemed to have a poor response. A universally accepted, clear-cut definition offers a means to effectively identify and address cases of suboptimal TKA outcomes, facilitating targeted interventions and action plans to improve overall patient care.

Furthermore, the global adoption of definitions requires that they are both valid (adequately reflects ‘poor response one year after TKA’) and feasible (easy to use and assess worldwide). The primary aim of this study was to seek consensus among international orthopedic knee experts regarding the face validity and feasibility of existing and newly proposed definitions for characterizing poor response one year after primary TKA. The secondary aim was to prioritize these definitions to identify those most crucial for assessing poor response to TKA, warranting further investigation.

## Materials and methods

This three-round online modified Delphi study is reported in line with recommendations for the Conducting and REporting of DElphi Studies (CREDES) [9, 19] and proposed Delphi study quality indicators [6].

### Project team and expert advisory group

A project team was formed to conduct the study comprising two orthopedic knee surgeons from the Netherlands and Belgium (JS, SvO), two researchers with backgrounds in rheumatology and orthopedics (CvdE, PH) and a PhD student (MtM). An expert advisory group, involving the five project team members and four international key experts with expertise and scientific publications on the measurement of outcomes after TKA, was established. The four key experts included a professor, orthopedic surgeon from Sweden (OR), a professor, rheumatologist from the USA (JS), a professor, epidemiologist and nurse from Australia (MD) and a leading orthopedic surgeon and orthopedic engineer from the UK (AP). Members of the expert advisory group were not members of the Delphi panel.

### Advisory group meetings

**Fig. 1:**
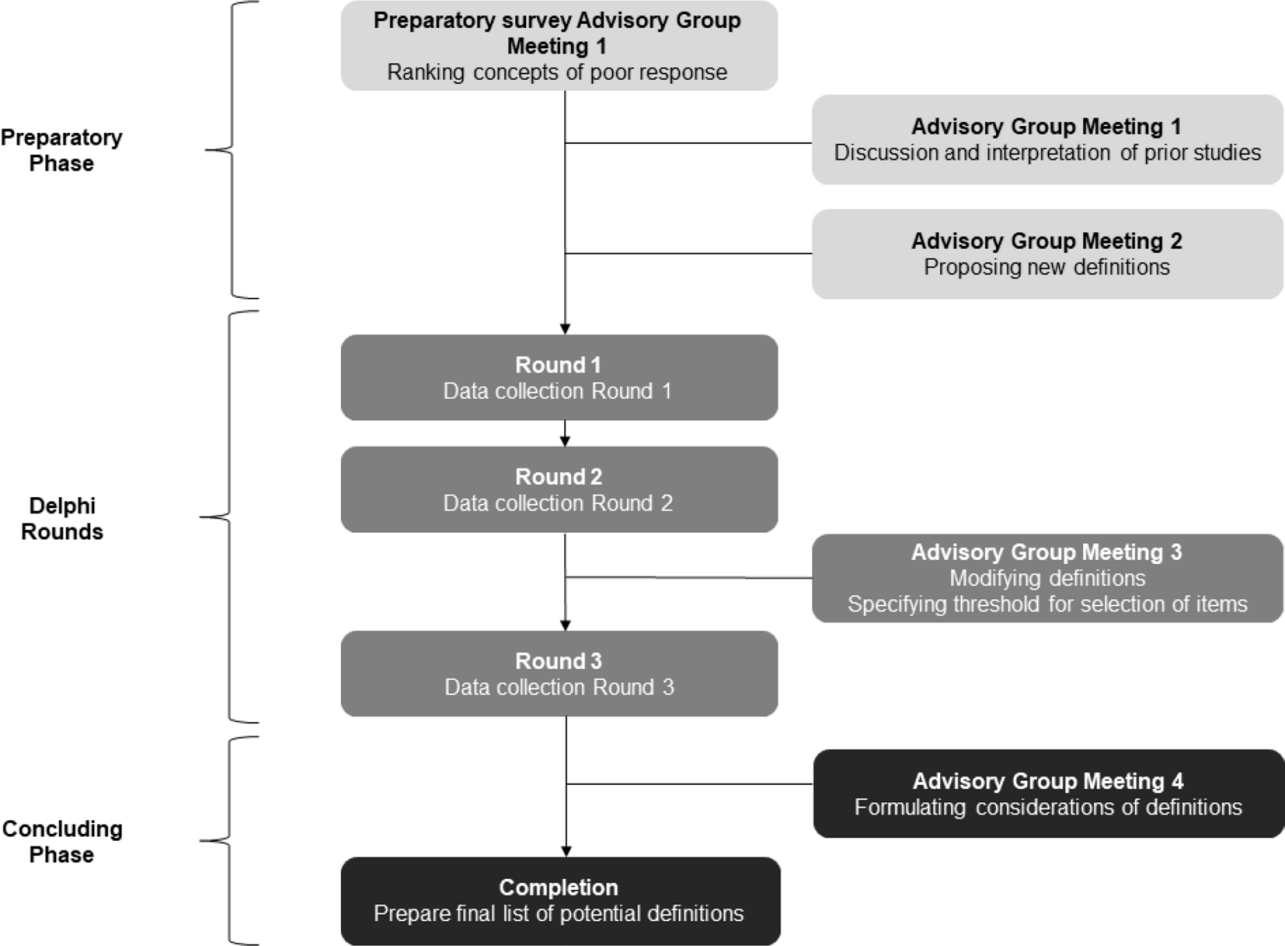
Flowchart of the Delphi process

The project team and expert advisory group met four times during the study (Fig. 1). All members were tasked with completing a survey featuring questions on their views regarding the importance of various domains and the nature of the threshold such as absolute cut-off value, absolute change, or relative change). The initial list of definitions for round 1 was created, based on a discussion of the results of the survey and previous studies of our research group [15–17]. During the third meeting, adjustments of definitions were discussed based on comments that arose from the first two Delphi rounds. Furthermore, the expert advisory group unanimously agreed on a median score lower than 6.5 as the threshold for the removal of definitions for the final round. The final meeting was organized to discuss the results of the Delphi exercise.

### Expert panel

There are no established guidelines on the optimal Delphi study panel size [11]. Therefore, a target of 50 panelists from at least 5 different countries worldwide, was set to ensure that international key stakeholders were sufficiently represented. Panelists were invited based on their recognized knowledge of the topic, their willingness to participate, and their intention to commit to the process.

Potential panelists were invited via e-mail to participate and were asked to nominate additional potential panelists (snowball sampling) [13]. To ensure clinical and research expertise, they were included in the panel when they met the following main eligibility criteria: (1) professional background as an orthopedic knee surgeon or orthopedic researcher; (2) (co)authored at least two publications on the outcome of TKA and/or performed at least 30 knee arthroplasties yearly.

### Delphi procedure

The procedure was performed between April and August 2021. It was decided a priori to include three rounds to increase convergence whilst minimizing participant attrition [18] (Fig. 1). All three surveys were hosted using SurveyMonkey [14] and administered via e-mail. All panelists who completed round 1 were subsequently emailed links to rounds 2 and 3. Panelists remained anonymous and unknown to each other throughout the entire process.

### Data collection

The initial draft list of definitions was provided to the Delphi panel (Online Resource 1). Panelists were asked to score the face validity (the degree to which the definition is an adequate reflection of ‘poor response one year after TKA’) and feasibility (the degree to which the definition is easy to use and assess worldwide) of each definition on a scale of 0 (very low face validity or not feasible) to 10 (very high face validity or feasible) and to justify each score. Free-text options were included at the end of the survey to allow panelists to suggest new definitions of poor response to TKA.

**Fig. 2:**
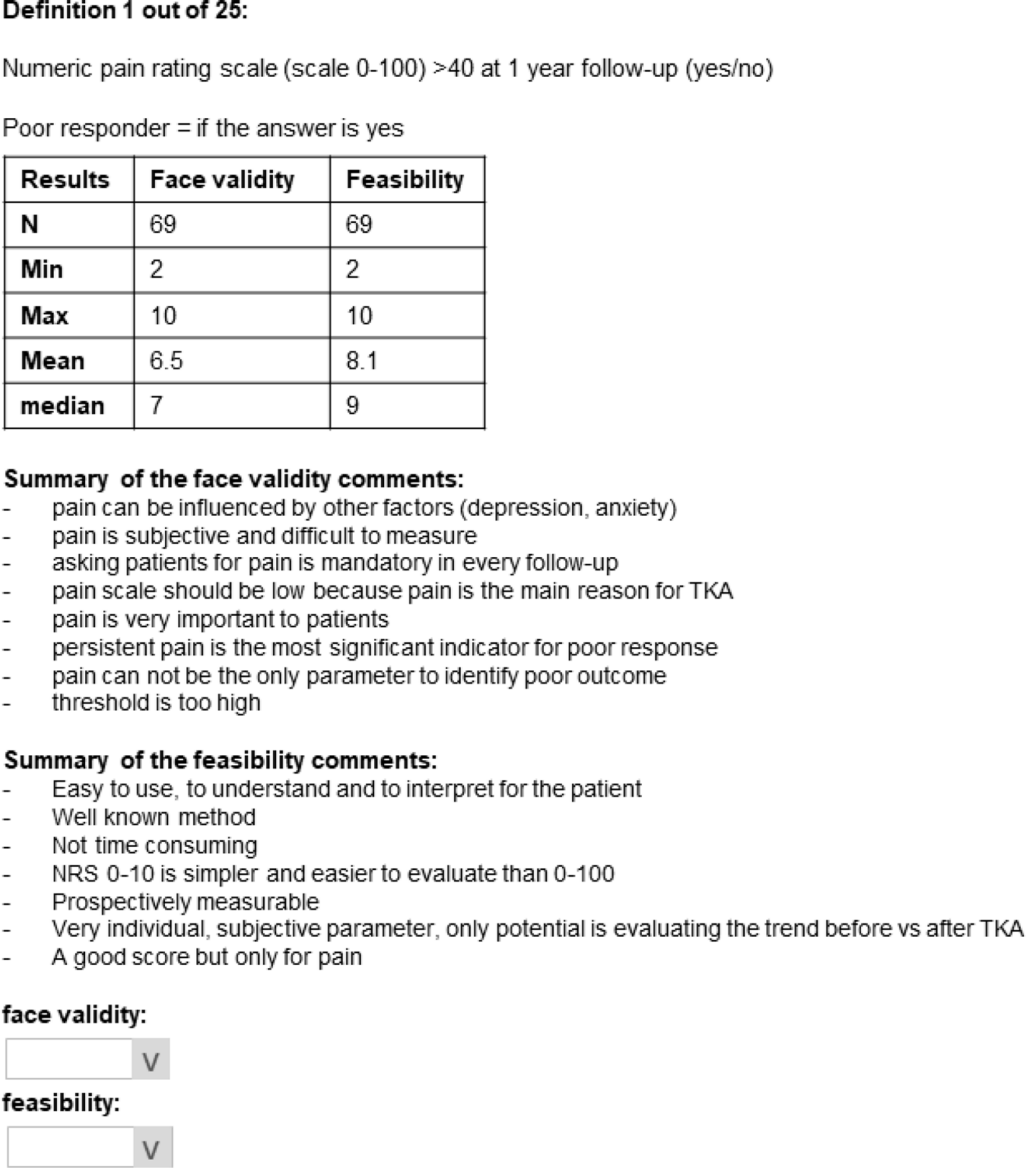
Round 2 survey item example

Round 2 featured tables for each definition from Round 1, displaying the number of panelists, metrics for face validity and feasibility, and a summary of comments made by panelists in Round 1. Additionally, Round 2 introduced newly suggested definitions generated from Round 1’s free-text responses. Panelists were prompted to reassess their ratings of Round 1 definitions and to score the face validity and feasibility of the new suggestions (on a scale of 0 to 10) (Fig. 2).

In round 3, definitions with a median score for face validity lower than 6.5 were removed from the list of definitions. Panelists were asked to distribute 100 points over the remaining definitions (*n* = 17) to rank the definitions of poor response.

### Data analysis

#### Qualitative analysis

A list with new definitions suggested by panelists (round 1) and a list of summarized comments (rounds 1 and 2) was discussed within the expert advisory group to add new definitions in round 2 and adapt the wording of definitions between rounds if considered necessary.

#### Quantitative analysis

The mean (SD) face validity and feasibility scores, the sum score of face validity plus feasibility (mean face validity score plus mean feasibility score), the ranking of definitions (total ranking points per definition) and the percentage of panelists that scored at least 1 point for a definition were analyzed descriptively using Microsoft Excel 365 and STATA 13.0 (StataCorp, College Station, TX).

## Results

### Panelists response

**Fig. 3:**
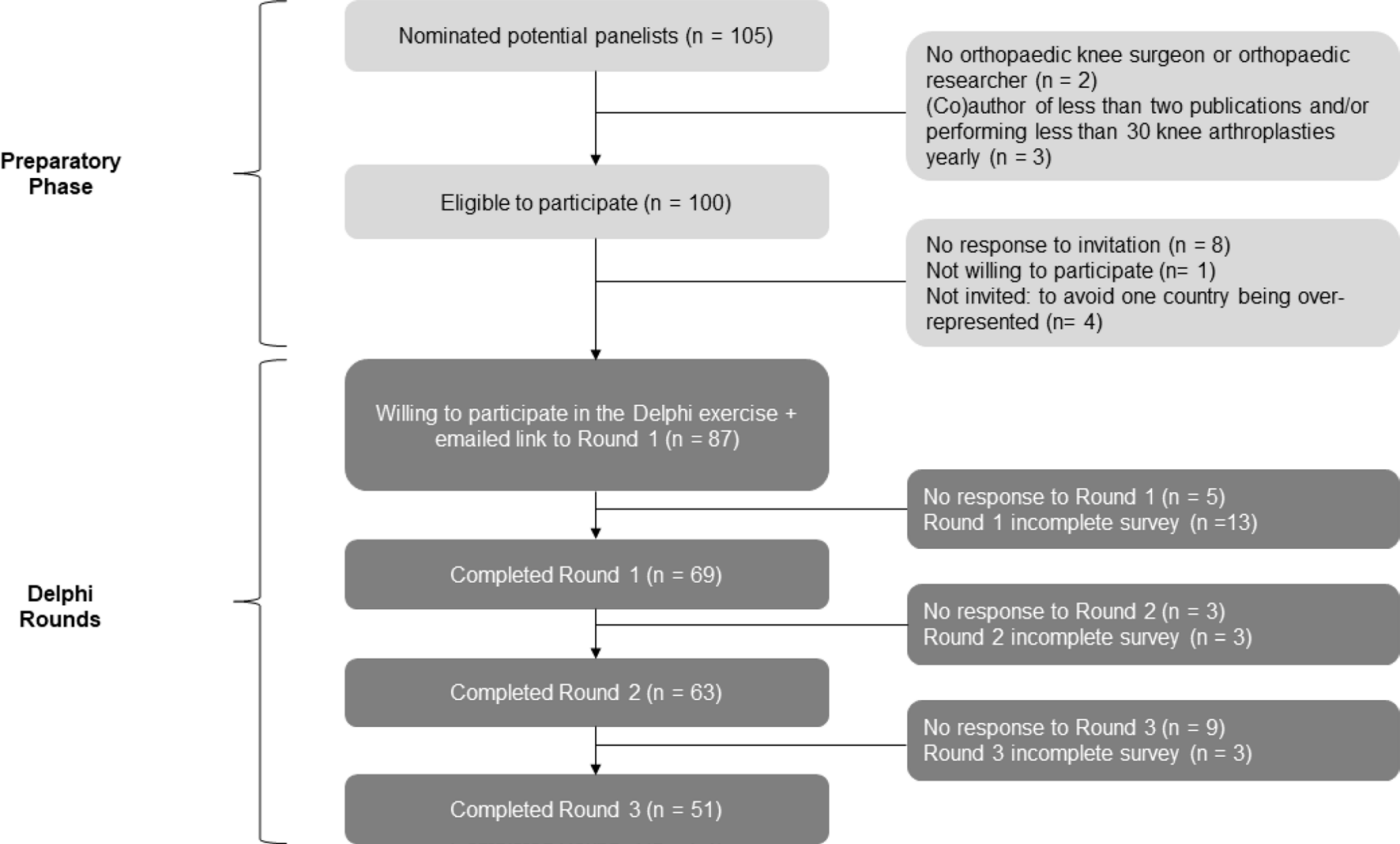
Flowchart of panelists

105 potential panelists were nominated and screened of whom 87 were found to be willing to participate (Fig. 3). A total of 69 panelists completed round 1 and formed the Delphi panel. Rounds 2 and 3 were completed by 63 (91%) and 51 (74%) panelists, respectively. Reasons for non-response of the different rounds were not available. The vast majority of the panelists were male, predominantly originating from Western European countries (Table 1).

**Table 1:** Characteristics of 69 panelists who completed round 1 and formed the Delphi panel

	Number of panelists (%) (*n* = 69)
Women *(N(%))* Missing	6(8.7%) 1(1.4%)
*Country of residency (N(%))* Netherlands UK USA Australia Italy	13(18.8%) 9(13.0%) 7(10.1%) 6(8.7%) 4(5.8%)
South Africa	3(4.3%)
Germany	3(4.0%)
Belgium France Norway Austria Indonesia Denmark Other ^a^	3(4.3%) 3(4.0%) 2(2.9%) 2(2.9%) 2(2.9%) 2(2.9%) 11(15.9%) 1(1.4%)
Missing	
*Current professional role (N(%))*	
Clinician	39(56.5%)
Clinician & researcher Researcher Missing	21(30.4%) 8(11.6%) 1(1.4%)

^a^ Other include the following countries: Finland, Slovenia, Indonesia, Scotland, Switzerland, Canada, Sweden, New Zealand, Greece, Spain and India

### Round 1

Panelists took on average 19 min to complete Round 1. Face validity and feasibility scores of the initial 25 included definitions ranged from 5.4 to 7 and from 5.5 to 8.4, respectively (Online Resource 1). 25 Panelists proposed 29 new different definitions of which 9 were added to round 2 based on consensus among the members of the expert advisory group (Online Resource 1).

### Round 2

Panelists took on average 17 min to complete Round 2.

Scores for face validity and feasibility range from 4.9 to 7.5 and from 5.2 to 8.5 respectively (Table 2 and Online Resource 1). A total of 17 out of 34 definitions with a median score of at least 6.5 for face validity served as input for round 3 (Table 2). Based on comments of panelists and after discussion among members of the expert advisory group, some adjustments (e.g. ‘since before TKA’ instead of ‘since TKA’) were made in the wording of 7 definitions.

**Table 2:** Face validity and feasibility scores and ranking list of definitions of poor response to TKA after Delphi round 3

Ranking based on total ranking points			Delphi round 2			Delphi round 3	
Ranking	Definition	Underlying domain(s)	Face validity mean (SD)	Feasibility mean (SD)	Sum score Face validity + Feasibility	Total ranking points	*N* respondents with ≥ 1 point ^a^ (*n* = 51)
1	No improvement in pain OR daily knee functioning compared to pre-operative status^b, c,d^	pain, physical functioning	6.5 (1.9)	7.5 (1.5)	14	544	46 (90.2%)
2	Single-item question on satisfaction with the outcome (scale: very unsatisfied - very satisfied) Poor responder = very unsatisfied, unsatisfied	satisfaction	7.5 (1.5)	8.5 (1.2)	16	497	47 (92.2%)
3	Single item-questions: No improvement in pain compared to preoperative status^b, c^	pain	7.4 (1.7)	8.1 (1.4)	15.5	386	44 (86.3%)
4	Single item question: “Considering your outcome, are you happy that you had your TKA surgery?” (scale: yes/no) Poor responder = if no	satisfaction	7.1 (2.2)	8.1 (1.6)	15.2	385	43 (84.3%)
5	Single item question: No improvement in daily knee functioning compared to pre-operative status (rising from sitting, walking, stair climbing)^b, d^	physical functioning	6.6 (1.7)	7.5 (1.7)	14.1	324	43 (84.3%)
6	Single item question on willingness to do TKA surgery again (yes/no) Poor responder = if no	satisfaction	6.9 (2.3)	8.2 (1.7)	15.1	320	40 (78.4%)
7	OKS pain & functioning (scale: 0–48) absolute improvement ≤ 6	pain, physical functioning	6.8 (1.4)	6.8 (1.6)	13.6	283	34 (66.7%)
8	OMERACT-OARSI responder criteria (WOMAC pain & functioning and global score): Non-responder: (< 50% improvement and < 20 absolute change in either pain or function) OR (no improvement in at least 2 of the 3 following: <20% improvement and < 10 absolute change in either pain, function or patient’s global assessment)	pain, physical functioning, global assessment	6.3 (1.8)	4.8 (1.7)	11.1	278	38 (74.5%)
9	Single item question on fulfillment of TKA expectations (scale: to a great extent - not at all) Poor responder = very little, not at all	satisfaction	6.9 (1.6)	7.5 (1.5)	14.4	276	38 (74.5%)
10	OKS PASS < 30 (scale: 0–48) PASS: Patient Acceptable Symptom State	pain, physical functioning	6.5 (1.7)	6.6 (1.8)	13.1	257	38 (74.5%)
11	NRS pain > 40 in the treated knee (scale: 0-100)	pain	6.8 (1.5)	8.0 (1.5)	14.8	255	40 (78.4%)
12	OKS pain & functioning < 26 (scale: 0–48)	pain, physical functioning	6.9 (2.0)	6.7 (1.9)	13.6	244	38 (74.5%)
13	Single item-question: No improvement in knee functioning during moderate activities (gardening, shopping, cycling)^b, d^	physical functioning	6.5 (1.7)	7.4 (1.6)	13.9	235	41 (80.4%)
14	OKS pain & functioning (scale: 0–48) absolute improvement ≤ 5	pain, physical functioning	7.1 (1.5)	6.9 (1.6)	14	234	36 (70.6%)
15	WOMAC pain, stiffness & functioning (scale: 0-100) absolute improvement < 10	pain, knee function, physical functioning	6.1 (1.6)	5.2 (1.7)	11.3	214	37 (72.5%)
16	New KSS symptoms subscale (scale: 0–25) absolute improvement < 15	pain	6.2 (1.5)	6.2 (1.7)	12.4	211	34 (66.7%)
17	Single item question on nocturnal knee pain causing sleep disturbance (yes/no) Poor responder = if yes	pain	7.1 (2.3)	8.4 (1.6)	15.5	157	32 (62.7%)

The ranking of definitions is based on total ranking points (column 7)

^a^ The number of panelists (%) that voted with at least 1 point for a definition

^b^ Specification of definitions based on transition question(s): How have your pain symptoms or daily knee functioning changed since your TKA?

^c^ Transition questions on change in pain and daily knee functioning range of 1 to 7, with 1 representing very deteriorated and 7 representing very improved. A score < 4 was categorized as poor response’;

^d^ Transition question on how daily knee functioning or functioning during moderate activities changed

(scale: much worse - much better) Poor responder = much worse, worse, a little worse, unchanged

KSS: Knee Society Score, NRS: Numeric Rating Scale, OKS: Oxford Knee Score, OMERACT-OARSI: Outcome Measures in Arthritis Clinical Trials-Osteoarthritis Research Society International, PASS: Patient Acceptable Symptom State, SD: Standard Deviation, WOMAC: Western Ontario and McMaster Universities Osteoarthritis Index

### Round 3

Panelists took on average 9 min to complete Round 3. The definition “No improvement in pain OR daily knee functioning compared to pre-operative status^”^ was the most highly prioritized (Table 2). The definition comprising a single item of (dis)satisfaction with the outcome of TKA was prioritized secondly and obtained the highest scores for face validity and feasibility (7.5, and 8.5 respectively).

## Discussion

This study is the first to identify and prioritize definitions that may identify poor response one year after TKA. The definition “No improvement in pain OR daily knee functioning compared to pre-operative status” was highest prioritized whereas the single-item definition on patient satisfaction with the outcome had the highest scores on face validity and feasibility. In general, panelists preferred single-item questions reflecting change compared to pre-operative status above definitions requiring pre- as well as post-surgery assessment of validated questionnaires.

Remarkably, the single-item definition of patient (dis)satisfaction with the outcome of TKA scored highest for face validity and feasibility suggesting that poor response after TKA is best reflected in this overarching concept. However, the concept “(dis) satisfaction” contrasts with indicators for TKA surgery, i.e. severe pain, and functional limitations, corroborated by radiographic findings [4, 7]. Our results suggest that the concept of patient dissatisfaction may capture more than only pain and daily functioning and better reflects “poor response” according to the panelists. There is a widely reported variation in dissatisfaction rates [5], and this variation may in part be explained due to the format of the question [2, 10], (e.g. yes/no format, and dichotomized Likert scales or Numeric Rating Scales with variable cut-offs) [5, 15]. Moreover, in general, single-item questions [15] are being used because validated patient (dis)satisfaction questions with standardized response options are scarce [2]. Clement et al. previously demonstrated that the wording of the satisfaction question significantly influences the rate of patient satisfaction one year after TKA [2]. However, despite the highest face validity and feasibility, the definition of patient (dis)satisfaction with the outcome was ranked second, likely due to the complexity associated with interpreting patient (dis)satisfaction.

The prioritized list also contains several definitions that include a predefined minimal difference. However, definitions containing a predefined difference received a lower ranking than definitions based on transition questions. A possible explanation for this is that the change in PROM scores depends on the patient initial baseline status [3], and thus requires preoperative as well as postoperative assessment of PROMs. On the other hand, definitions based on transition questions (including questions on (dis)satisfaction) are subject to recall bias, because patients might not remember their preoperative conditions adequately one year after the procedure.

Prioritized definitions in this study mainly describe change from the patient’s perspective on underlying domains such as pain, physical functioning, and satisfaction (Table 2). It is noteworthy that the list of ranked definitions does not contain clearly defined, more objective elements as knee flexion < 90⁰, flexion contracture > 10⁰ or revision surgery within one year after the initial procedure, despite the inclusion of such objective measures in the initial list of definitions. This finding implies that researchers and clinicians place greater emphasis on subjective measures from the patient’s perspective rather than relying solely on objective measures or the clinical judgment of clinicians.

Perhaps unsurprisingly, the single-item definition on satisfaction with the outcome of TKA received the highest feasibility score. Cost-free availability and brevity make this definition feasible to measure poor response to TKA. However, it is important to acknowledge that this definition serves as a crude indicator, offering abstract information. While this may be adequate for clinical practice as a starting question to elicit problems, it may not provide sufficient detail for research purposes and quality improvement. Further research is necessary to recommend specific definitions separately for research purposes and clinical practice as the balance between feasibility and face validity may differ between clinical practice and research settings. A prospective, longitudinal study would be of interest to compare the ability of definitions to discriminate between patients having a poor response and those without.

Another conclusion that can be drawn from the prioritized list is that the high-prioritized definitions do not include validated PROMs and received lower scores for feasibility. Feasibility considerations of panelists and members of the expert advisory group indicate that an international definition should not depend on previously validated questionnaires as these are not available in all languages and are not easy to assess worldwide and in clinical practice. Furthermore, the volume of questions in PROMs can easily become burdensome. A possible explanation is that validated PROMs are not (yet) feasible for clinical practice or benchmarking but more suitable for research purposes.

### Strengths & limitations

The strength of a web-based survey is that it ensured anonymity between panelists, which minimizes social pressures and avoids group decisions being dominated by specific experts [9]. Remote data collection facilitated inclusion of a broad range of international key experts in the orthopedic field, with at least 23 different countries being represented.

The main limitation of the present study might be a suboptimal representation of the expert advisory group and Delphi panel, as it did not involve TKA patients or other stakeholders (e.g. allied health practitioners). We deliberately chose not to include patient representatives in this study considering the need for strong English language skills due to the international nature of the study, as well as the complexity associated with the Delphi exercise itself. However, we processed patient input from the previous interview study and decided to perform a separate study on the prioritization of adverse consequences of TKA among patients.

Another limitation is that several panelists indicated that they were not familiar with certain PROMs or metrics (e.g., MCID, PASS: Patient Acceptable Symptom State) used in the definitions, which could have affected the assessment and ranking of definitions. Finally, despite our comprehensive efforts to recruit panelists from around the world, there was under-representation of several continents. Most of the panelists worked in a European country, North America, or Australia, which may limit the generalizability of the findings. The main contributing factor to this is that the Delphi panel was set up by the members of the expert advisory group working on these continents.

## Conclusions

This study with representation from 23 countries across the globe is the first to attempt to define poor response to TKA. We identified seventeen potential definitions. The definition “No improvement in pain OR daily knee functioning compared to pre-operative status” was highest prioritized whereas the single-item definition on patient satisfaction with the outcome had the highest scores on face validity and feasibility. Remarkably, none of the definitions are based on the assessment of knee function by the clinician and none are complication, surgery- or revision-related. Our findings can guide future quality improvement efforts to identify patients with a poor response to TKA.

## Electronic supplementary material

Below is the link to the electronic supplementary material.

Supplementary Material 1

## Data Availability

The datasets used and/or analysed during the current study are available from the corresponding author on reasonable request.
