# Supplementary material for: International consensus-based ranking of definitions for poor response to primary total knee arthroplasty: a Delphi study
Source: Arch Orthop Trauma Surg. 2024 Sep 11;144(11):4697–706. doi: 10.1007/s00402-024-05515-y (PMC11582167; doi:10.1007/s00402-024-05515-y)
Supplement: Supplementary file 1 — Supplementary Material 1 [file 402_2024_5515_MOESM1_ESM.docx]

**International consensus-based ranking of definitions for poor response to primary total knee arthroplasty: A Delphi study**

**Supplementary Table 1: Complete overview of face validity and feasibility scores of Delphi round 1 and 2.**

**This table includes definitions from the initial draft list (indicated by white rows) and new definitions generated from panelists’ free-text responses in Round 1, which were subsequently included in the Round 2 survey (indicated by light-shaded rows).**

| **Definitions Delphi Round 1 and 2** | **Round 1** | | **Round 2** | |
| --- | --- | --- | --- | --- |
|  | **Face validity mean (SD)** | **Feasibility**  **mean (SD)** | **Face validity mean (SD)** | **Feasibility**  **mean (SD)** |
| NRS pain >40 (scale: 0-100) | 6.5 (1.9) | 8.1 (2.0) | 6.8 (1.5) | 8.0 (1.5) |
| IKSS pain <30 (scale: 0-50) | 6.4 (2.1) | 7.0 (2.1) | 6.2 (1.5) | 6.2 (1.7) |
| Knee flexion <90° | 5.9 (2.4) | 7.5 (2.4) | 5.9 (2.1) | 7.3 (1.7) |
| IKSS functioning <60 (scale: 0-100) | 6.2 (2.0) | 6.9 (2.0) | 5.8 (1.4) | 6.1 (1.6) |
| Single item question: “Have you been able to return to the activity (or activities) that your knee stopped you from doing one year ago?”  (scale: yes/no) Poor responder = if no | 5.4 (2.5) | 7.2 (2.3) | 5.1 (1.6) | 6.7 (1.7) |
| Single item question on satisfaction with the outcome (scale: very unsatisfied - very satisfied)  Poor responder = very unsatisfied, unsatisfied | 7.4 (2.3) | 8.4 (1.8) | 7.5 (1.5) | 8.5 (1.2) |
| OKS pain & functioning (scale: 0-48) absolute improvement ≤5 | 7.1 (1.8) | 7.6 (1.9) | 7.1 (1.5) | 6.9 (1.6) |
| OKS pain & functioning (scale: 0-48) absolute improvement ≤6 | 6.7 (1.9) | 7.4 (2.0) | 6.8 (1.4) | 6.8 (1.6) |
| OMERACT-OARSI responder criteria (WOMAC pain & functioning and global score): Non-responder: (<50% improvement and <20 absolute change in either pain or function) OR (no improvement in at least 2 of the 3 following: <20% improvement and <10 absolute change in either pain, function or patient’s global assessment) | 6.6 (2.4) | 5.5 (2.5) | 6.3 (1.8) | 4.8 (1.7) |
| WOMAC pain, stiffness & functioning (scale: 0-100) absolute improvement <7.5 | 6.2 (2.3) | 6.6 (2.3) | 6.1 (1.6) | 5.2 (1.7) |
| < 50% improvement and an absolute change of < 20 in pain scale 0-100  (yes/no) Poor responder = if yes | 6.1 (2.0) | 6.7 (2.2) | 5.7 (1.7) | 6.4 (1.9) |
| No improvement on transition question on change in pain^1^ | 7.1 (2.3) | 8.2 (2.0) | 7.4 (1.7) | 8.1 (1.4) |
| < 30% improvement and an absolute change of < 10 in knee functioning scale 0-100  (yes/no) Poor responder = if yes | 5.2 (2.3) | 6.1 (2.5) | 5.3 (1.7) | 5.8 (1.6) |
| No improvement on transition question on change in daily knee functioning (rising from sitting, walking, stair climbing)^2^ | 6.7 (2.1) | 7.8 (1.9) | 6.6 (1.7) | 7.5 (1.7) |
| No improvement on transition question on change in knee functioning during moderate activities (gardening, shopping, cycling)^2^ | 6.5 (2.2) | 7.8 (2.0) | 6.5 (1.7) | 7.4 (1.6) |
| Composite question: “Have you had any serious complication (e.g. infection) that has required further surgery or revision of the prosthesis?” (scale: yes/no)  OR  “Has your knee been replaced?” (scale: yes/no)  Poor responder = if yes on one or both questions | 5.9 (3.2) | 7.6 (2.6) | 5.2 (2.4) | 7.7 (1.8) |
| < 50% improvement and an absolute change of < 20 in pain scale 0-100 (yes/no)  OR  < 30 % improvement and an absolute change of < 10 in knee functioning scale 0-100 (yes/no)  Poor responder = if yes on one or both questions | 5.5 (2.2) | 5.8 (2.3) | 5.4 (1.6) | 5.4 (1.8) |
| No improvement on transition question on change in pain OR daily knee functioning^1^ | 6.8 (2.3) | 7.6 (2.2) | 6.5 (1.9) | 7.5 (1.5) |
| < 50% improvement and an absolute change of < 20 in pain scale 0-100 (yes/no)  OR  No improvement on transition question on change in pain OR daily knee functioning^1,2^  Poor responder = if yes on the first question or much worse, worse, a little worse on the second question | 5.7 (2.2) | 6.7 (2.0) | 5.8 (1.7) | 6.2 (1.5) |
| < 30 % improvement and an absolute change of < 10 in knee functioning scale 0-100 (yes/no)  OR  No improvement on transition question on change in pain OR daily knee functioning^1,2^  Poor responder = if yes on the first question or much worse, worse, a little worse on the second question | 5.6 (2.1) | 6.5 (2.1) | 5.4 (1.7) | 6.1 (1.5) |
| < 20% improvement and an absolute change of < 10 in pain (yes/no)  OR  < 20% improvement and an absolute change of < 10 in knee functioning (yes/no)  AND  No improvement on transition question on change in pain OR daily knee functioning^1,2^  Poor responder = if yes on one of the first two questions and much worse, worse, a little worse on the third question | 5.6 (2.5) | 6.2 (2.6) | 5.4 (2.1) | 5.6 (1.9) |
| < 50% improvement and an absolute change of < 20 in pain (yes/no)  OR  How happy/satisfied are you with the level of improvement in your pain?  Poor responder = if yes on the first question or very unsatisfied, unsatisfied on the second question | 5.8 (2.2) | 6.7 (2.4) | 5.9 (1.9) | 6.2 (1.8) |
| < 50% improvement and an absolute change of < 20 in pain (yes/no)  OR  How happy/satisfied are you with the level of improvement in your pain?  OR  How happy/satisfied are you with the level of improvement in your knee functioning?  Poor responder = if yes on the first question or much worse, worse, a little worse on the second question or very unsatisfied, unsatisfied on the third question | 5.7 (2.3) | 6.3 (2.5) | 5.4 (2.0) | 5.5 (1.9) |
| < 30% improvement and an absolute change of < 10 in knee functioning (yes/no)  OR  How happy/satisfied are you with the level of improvement in your knee functioning?  Poor responder = if yes on the first question or very unsatisfied, unsatisfied on the second question | 5.6 (2.2) | 6.5 (2.3) | 5.3 (1.9) | 5.7 (1.8) |
| < 30% improvement and an absolute change of < 10 in knee functioning (yes/no)  OR  How happy/satisfied are you with the level of improvement in your pain?  OR  How happy/satisfied are you with the level of improvement in your knee functioning?  Poor responder = if yes on the first question or very unsatisfied, unsatisfied on the second and third question | 5.7 (2.3) | 6.4 (2.2) | 5.2 (2.0) | 5.5 (1.9) |
| Single item question: "Considering your outcome, are you happy that you had your TKA surgery?" (scale: yes/no)  Poor responder = if no |  |  | 7.1 (2.2) | 8.1 (1.6) |
| Lack of extension > 10° (scale: yes/no)  Poor responder = if yes |  |  | 5.2 (2.5) | 6.6 (2.3) |
| Single item question on willingness to do TKA surgery again (scale: yes/no)  Poor responder = if no |  |  | 6.9 (2.3) | 8.2 (1.7) |
| Single item question on fulfillment of TKA expectations (scale: to a great extent - not at all)  Poor responder = very little, not at all |  |  | 6.9 (1.6) | 7.5 (1.5) |
| OKS PASS <30 (scale: 0-48)  PASS: Patient Acceptable Symptom State |  |  | 6.5 (1.7) | 6.6 (1.8) |
| OKS pain & functioning <26 (scale: 0-48) |  |  | 6.9 (2.0) | 6.7 (1.9) |
| Composite question: “Are you dissatisfied with either pain OR function? (scale: yes/no)  Poor responder = if yes for one or both questions |  |  | 6.2 (2.1) | 6.8 (2.1) |
| Single item question on nocturnal knee pain causing sleep disturbance (scale: yes/no)  Poor responder = if yes |  |  | 7.1 (2.3) | 8.4 (1.6) |
| Single item question: “Are you aware of your knee every day?” (yes/no)  Poor responder = if yes |  |  | 4.9 (2.1) | 7.0 (2.1) |
| KSS: Knee Society Score, NRS: Numeric Rating Scale, OKS: Oxford Knee Score, OMERACT-OARSI: Outcome Measures in Arthritis Clinical Trials-Osteoarthritis Research Society International, PASS: Patient Acceptable Symptom State, SD: Standard Deviation, WOMAC: Western Ontario and McMaster Universities Osteoarthritis Index  ^1^ Transition questions on change in pain and daily knee functioning range of 1 to 7, with 1 representing very deteriorated and 7 representing very improved. A score <4 was categorized as poor response’;  ^2^ Transition question on how daily knee functioning or functioning during moderate activities changed, (scale: much worse - much better) Poor responder = much worse, worse, a little worse, unchanged | | | | |
